# Wound Leakage With the Use of Calcium Sulphate Beads in Prosthetic Joint Surgeries: A Systematic Review

**DOI:** 10.7759/cureus.19650

**Published:** 2021-11-16

**Authors:** Muhammad Yasir Tarar, Aizaz Khalid, Muhammad Usman, Komal Javed, Numan Shah, Muhammad Waqar Abbas

**Affiliations:** 1 Trauma and Orthopaedics, Salford Royal NHS Foundation Trust, Manchester, GBR; 2 Internal Medicine, Services Institute of Medical Sciences, Lahore, PAK; 3 Plastic Surgery, St George's University, London, GBR; 4 Accident and Emergency, Glangwili General Hospital, Carmarthen, GBR

**Keywords:** wound leakage, total hip replacement (thr), total knee replacement (tkr), pji, calcium sulphate beads

## Abstract

Since its first use as a bone void filler at the end of the 19th century, calcium sulphate products have been adapted in different ways to aid orthopaedic surgeons. Calcium sulphate local antibiotic delivery systems offer a promising solution in the delivery of high antibiotic concentrations locally for an extended period of time. Over the years, multiple centres have reported side effects such as wound drainage, heterotrophic ossification and hypercalcaemia. This study was carried out to assess the risk of wound drainage in prosthetic joints after implantation of antibiotic-impregnated calcium sulphate beads. Two reviewers searched the literature in three online databases using the Cochrane methodology for systematic reviews. The search of databases yielded 182 articles. The studies without reported post-operative complications, mainly drainage outcomes, were excluded. After screening, seven articles were deemed suitable and selected. Out of the 1,112 cases identified, 43 joints developed wound drainage after calcium sulphate bead placement. This complication was resolved in all these cases by either conservative or operative approaches. The factors implicated in the development of wound drainage include the volume of the product used, procedural placement and host factors. The result of this systematic review shows that calcium sulphate products can be used for treatment and prophylaxis in prosthetic joints with a risk of post-procedural wound drainage. This risk, however, is lesser with the use of synthetic calcium sulphate products as compared with conventional calcium sulphate products.

## Introduction and background

In England and Wales, there are about 160,000 joint replacements performed in a year [1]. This number is up to one million in the United States [2,3] and is expected to increase to four million a year by 2030 [4]. In recent times, calcium sulphate beads have been effectively used in septic and aseptic conditions. The incidence of prosthetic joint infection (PJI) is 1%-2% after primary replacement, with a weighted mean of 0.97% in total hip replacement (THR) and 1.03% in total knee replacement (TKR) from multiple national registries [5]. Prosthetic joint infection (PJI) is a serious limb and life-threatening complication of joint replacement surgery. The current protocol for PJI includes debridement and antibiotic therapy. Surgeons have been increasingly using calcium sulphate beads to deliver antibiotics to the joint space. Calcium sulphate beads are traditionally used as bone filling agents [6]. However, they have been found to be effective in treating PJI as the beads are completely absorbed from the joint space and therefore do not need to be surgically removed. Moreover, because of the beads getting absorbed, 100% of the antibiotic load is released [7].

There is limited literature on the risk associated with using calcium sulphate beads; however, one reported developing wound drainage as a risk of using calcium sulphate beads. The incidence of wound drainage is reported to range from up to 51% [8]. The consequences of wound drainage include a longer hospital stay for the patient, wound care and immobilisation. If wound drainage persists, washout and closure may be indicated. Persistent wound drainage may increase the risk of infection [9]; however, Ferguson et al. found no infections in those treated non-surgically for wound drainage [10]. Wound drainage has been associated with higher volumes of calcium sulphate beads, particularly volumes greater than 20 cc [11]. Wound drainage is also linked to a deeper subcutaneous insertion site and a McPherson systemic host score of C [12].

The aim of this study is to determine the risk of developing wound drainage when using calcium sulphate beads in any prosthetic joint surgery. We aim to determine the factors that increase the risk of developing wound drainage and what measures can be taken to treat wound drainage.

## Review

Methodology

**Figure 1:**
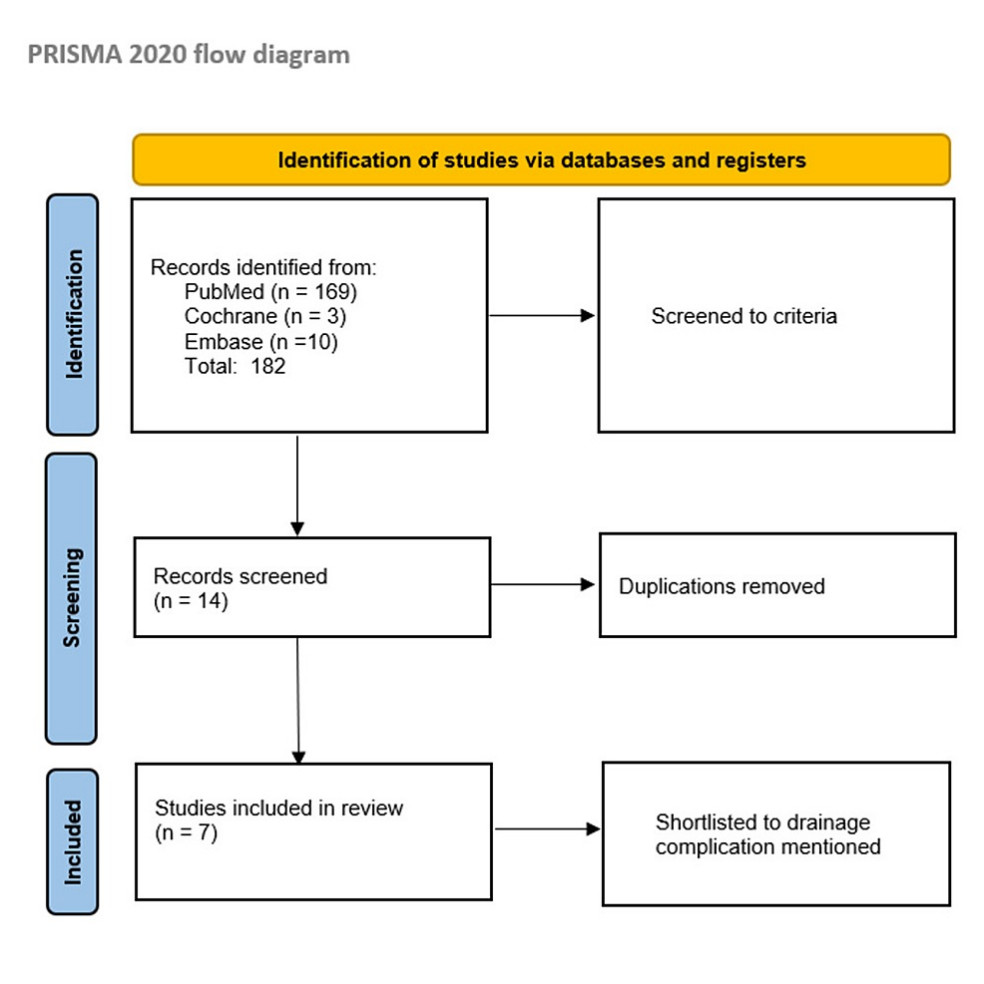
Study methodology used for the systematic review (PRISMA). PRISMA: Preferred Reporting Items for Systematic Reviews and Meta-Analyses

A literature search of three online databases, namely, MEDLINE (1946 to present), Embase (1974 to present) and Cochrane CENTRAL (1988 to present), using the Cochrane methodology for systematic review was conducted using the keywords 'prosthetic joint', 'total knee replacement' and 'total hip replacement' and cross-searched with 'calcium sulphate beads'. The inclusion criteria selected was any study that has reported complications in any prosthetic joint surgery regardless of the indication and regardless of the language in which the study was published. Studies without reported post-operative drainage outcomes were excluded. All full texts were retrieved and reviewed where open access was not available. Search screening and article shortlisting were performed using the Preferred Reporting Items for Systematic Reviews and Meta-Analyses (PRISMA) methodology (Figure 1) [13].

The search yielded a total of 182 articles. All shortlisted studies were reviewed by the junior and senior study members. After review, seven articles were selected in the study as per the inclusion criteria (Table 1). Data from the selected studies, including the type of calcium sulphate beads and the dose used, and drainage outcomes, are tabulated for a detailed review (Table 1).

**Table 1:** Type of calcium sulphate derivatives used in the joints, incidence of wound drainage and management. S/C: subcutaneous, G/A: general anaesthetic

Author	Patients with Prosthetic Joint Procedures (N)	Calcium Sulphate Bead Derivative and Dosage	Space Calcium Sulphate Beads Used In	Wound Closure After Calcium Sulphate Insertion	Wound Leakage	Management of Wound Leakage
Sandiford [17]	29	Stimulan, 20–40 mL (mean: 25 mL)	Intracapsular compartment of hip joints, none in S/C tissue	Capsule closed over beads	1/29 (3.4%)	Re-exploration under G/A + closure of soft tissue defect
Kallala and Haddad [18]	15	Stimulan, 10–40 mL	Around the hip/knee joints/prosthesis or spacer, none in S/C tissue	Wound closure methods not reported	0/15 (0%)	N/A
Kallala et al. [19]	755	Stimulan, 5–80 cc (mean: 23.39 cc)	Knees – medial and lateral gutters	Wound closure methods not reported	32/755 (4.2%)	Drainage >5 days post-operatively/serous/serosanguineous – wound redressed and anticoagulants stopped
			Hips – placed deeply, inferior to the acetabulum and around the proximal femur			Drainage >5 days post-operatively/sanguineous discharge – washout under G/A
McPherson et al. [20]	250	Stimulan, 5–50 cc	Knees – medial and lateral gutters, none in S/C tissue	Drain placed in the lateral gutter, wound closed in mid-flexion + - S/C drains	3.2% overall	Lavage, debridement, wound VAC placement and/or compressive dressing
					3.5% knee wound drainage	
		Stimulan, 5–70 cc	Hips – deep hip space, inferior to the acetabulum and around the proximal femur, none placed in S/C tissue	Drain placed under TFL layer + - S/C drains, tension-free closure	2.8% hip wound drainage	
Menon et al. [21]	3	Stimulan (volume not reported specifically for PJI cases)	Not reported	Not reported for PJI	1/3 wound patients had wound drainage	Re-exploration
Lum et al. [22]	56	Stimulan (volume not reported)	Not reported	Calcium sulphate beads placed into the wound during final closure	1/56 (1.7%)	Resolved without surgical intervention
Agarwal et al. [23]	4	Stimulan (maximum volume: 20 cc)	Within the joint space and medullary canal	Not reported	1/4 persistent culture negative discharge	Managed with regular dressing changes

Discussion

In our review, across seven studies, we identified 1,112 patients who underwent prosthetic joint surgeries for various aseptic and septic indications (Table 2) with calcium sulphate bead implantation. Out of these 1,112 cases, 43 (3.8%) joints developed wound drainage as a complication of calcium sulphate bead use. Out of the selected studies, 578 patients had a prosthetic joint infection, with 237 hip PJI and 341 knee PJI. Five out of the seven selected studies have specified drainage in the PJI cohort; however, two studies (Kallala et al. [19] and Lum et al. [22]) have not specified drainage complications in the PJI patient group (Table 3). The diagnosis of PJI is commonly made using the criteria proposed by the Musculoskeletal Infection Society (Table 4) [14]. A combination of conservative and operative strategies was reported by the authors, which eventually resulted in resolution in all cases.

**Table 2:** Demographics, antibiotic combinations and procedures performed with follow-up details of the selected articles. N: number of patients; THA: total hip arthroplasty

Author	Study Method	Study Type	Mean Age	Sex (Male:Female)	Antibiotics Used	Procedures Calcium Sulphate Beads Were Used In	Follow-Up
Sandiford [17]	Prospective	Case series	67	13:16	Vancomycin (n = 28)	Single-staged THA revision (n = 7)	Six weeks
						First-stage THA revision (n = 4)	
						Second-stage THA revision (n = 9)	
					Gentamicin (n = 27)	DAIR (n = 6)	
					Amikacin (n = 1)	Excision hip arthroplasty (n = 1)	
					Caspofungin (n = 1)	Periprosthetic hip fracture (n = 2)	
Kallala and Haddad [18]	Retrospective	Case series	64.8	8:7	Vancomycin + gentamicin (n = 15)	Single-stage revision (resurfacing arthroplasty to THR) (n = 2)	Mean 16 months (12–22)
						Primary THA revisions (n = 3)	
						Infected THA revisions (n = 3)	
						Proximal femoral replacement revision (n = 1)	
						Primary TKA revisions (n = 3)	
						Infected TKA revisions (n = 3)	
Kallala et al. [19]	Prospective	Case series	63	374:381	Vancomycin + tobramycin (n = 755)	TKA revision (n = 456)	Mean 35 months (0–78)
					(Amphotericin B included in fungal infections) (n = not reported)	THA revision (n = 299)	
McPherson et al. [20]	Retrospective	Case series	Not reported	Not reported	Vancomycin + tobramycin (n = 250)	Aseptic TKA revision (n = 66)	Minimum of three months for all patients (maximum of 12 months post-operatively)
						DECRA TKA (n = 16)	
						TKA resection (n = 35)	
						TKA re-implantation (n = 25)	
						Aseptic THA revision (n = 58)	
						DECRA THA (n = 8)	
						THA resection (n = 24)	
						THA re-implantation (n = 18)	
Menon et al. [21]	Retrospective	Case series	51	28:11	Vancomycin (n = 17)	Total prosthetic joint infections (n = 3), staged revision: debridement, biopsy +- implant removal	Minimum period of six months for all cases
					Colistin (n = 11)		
					Vancomycin + colistin (n = 8)		
					Vancomycin + gentamicin (n = 4)	Revision for infected TKR (n = 2)	
					Voriconazole (n = 1)	Revision for infected bipolar hemiarthroplasty (n = 1)	
Lum et al. [22]	Retrospective	Case series	Not reported	Not reported	Tobramycin + vancomycin + cefazolin (n = 56)	Primary TKA (n = 6)	Two weeks, six weeks, 12 weeks and one year
						Clean TKA revision (n = 12)	
						Infected TKA revision (n = 8)	
						Primary THAs (n = 5)	
						Clean THA revision (n = 19)	
						Infected THA revision (n = 6)	
Agarwal et al. [23]	Retrospective	Case series	Not reported	Not reported	Vancomycin (n = 3)	Single-stage TKR revision (n = 1)	Six weekly intervals, average follow-up 19 months
						Knee fusion using antegrade nail (n = 1)	
					Daptomycin (n = 1)	Single-stage hip revision (n = 1)	
						Cemented hip replacement (n = 1)	

**Table 3:** Wound leakage reported in patients with prosthetic joint infections in the selected studies.

Study Name	Prosthetic Joint infection	Hips (n)	Knees (n)	Leakage (Hips)	Leakage (Knees)
Sandiford [17]	29	29	0	1	0
Kallala and Haddad [18]	15	9	6	0	0
Kallala et al. [19]	387	140	247	Not specified in PJI (overall: 32 in 755)	
McPherson et al. [20]	126	50	76	2	0
Menon et al. [21]	3	1	2	0	0
Lum et al. [22]	14	6	9	Not specified in PJI (overall: 1 in 56)	
Agarwal et al. [23]	4	2	2	1	0

**Table 4:** Musculoskeletal Infection Society (MSIS) definition of prosthetic joint infection [14].

Prosthetic Joint Infection
1. There is a sinus tract communicating with the prosthesis; or
2. A pathogen is isolated by culture from at least two separate tissue or fluid samples obtained from the affected prosthetic joint; or
3. Four of the following six criteria exist:
1. Elevated serum erythrocyte sedimentation rate (ESR) and serum C-reactive protein (CRP) concentration,
2. Elevated synovial leukocyte count,
3. Elevated synovial neutrophil percentage (PMN%),
4. Presence of purulence in the affected joint,
5. Isolation of a microorganism in one culture of periprosthetic tissue or fluid, or
6. Greater than five neutrophils per high-power field in five high-power fields observed from histologic analysis of periprosthetic tissue at ×400 magnification.

While the efficacy of local antibiotic delivery by calcium sulphate beads is well documented, potential side effects have also been recognised, which include excessive wound drainage requiring intervention, heterotrophic calcification, reactive synovitis and transient hypercalcaemia. Chronic infections in prostheses are characterised by biofilms that are not easily eradicated by conventional antibiotic therapy [15]. Any residual biofilm after implant removal can serve as a focus for persistent infection and morbidity. Conventional methods including parenteral antibiotics fail to achieve the desired concentration in local tissue for reliable and effective eradication of infection. Local antibiotic delivery systems offer a promising solution to this problem. Kanellakopoulou et al. showed that an antibiotic-soaked calcium sulphate delivery system (Stimulan) can provide concentrations more than 300 times the minimum inhibitory concentration of causative organisms in studies on rabbits [16]. This method of delivery not only promises effective antimicrobial activity but also avoids the systemic toxicity of systemic antibiotics.

The pathophysiology of wound drainage due to calcium sulphate is well established but poorly understood. This adverse effect is thought to occur due to accelerated absorption of calcium sulphate beads, which then lead to the development of a calcium-rich fluid. This calcium-rich fluid is suspected to cause increased wound drainage via two possible mechanisms. Firstly, the calcium-rich hyperosmolar fluid exerts an osmotic effect and promotes fluid sequestration in the surgical planes where the beads have been planted. Secondly, it is thought to trigger a direct inflammatory response that leads to fluid collection [24,25]. Both these mechanisms may contribute to the wound drainage seen in patients after the use of calcium sulphate products. However, the exact mechanism is still unknown.

McPherson et al. proposed that this inflammatory process was due to the derivation of prior calcium sulphate products from gypsum, a naturally derived compound [20]. Stimulan, a commercially pure, synthetic calcium sulphate compound, offered a less harsh atmosphere to the synovial joint environment and was a promising answer to this problem [20]. This was reinforced by their study that showed that only 3.2% of the cases developed significant wound leakage that required intervention. Furthermore, they attributed these cases to excessive use of the product, quality of local tissue and health of the subjects. They recommended the use of Stimulan to be restricted to less than 30 cc to avoid this complication (Table 1) [20].

In cases where wound drainage was significant enough to warrant intervention, a variety of methods were employed. McPherson et al. used lavage with debridement wound VAC placement or application of compressive dressing, which resulted in eventual resolution [20]. Kallala et al. stopped anticoagulants if the drainage was found within five days with redressing or if it occurred after five days and thorough washout was done under general anaesthesia [19]. The single case of wound drainage in Sandiford's series was treated by re-exploration upon which a defect was found in the insertion of abductors, closure of which resulted in resolution [17]. Other studies reported resolution of drainage with conservative management such as redressing and observation [22,23].

Wound drainage is also thought to be related to the procedural placement of calcium sulphate beads. Subcutaneous placement is thought to lead to increased wound drainage and is avoided by watertight closure of deeper spaces [21]. McPherson et al. [20] and Kallala et al. [18,19] planted Stimulan beads in the medial and lateral gutters in knee arthroplasty. The deep hip space inferior to the acetabulum was used in the hip cases. Agarwal et al. used the bigger beads within the joint space with smaller bead insertion into the medullary cavity (Table 1) [23]. Subcutaneous positing of beads was actively avoided in all studies, which is also recommended by the manufacturers.

Several factors are implicated in the development of wound drainage after calcium sulphate bead use. These include the volume of beads used, their subcutaneous placement and the overall medical condition of the host. Variable volumes of Stimulan have been employed, ranging from 5 to 50 cc in knee cases and 5 to 70 cc in hip procedures by McPherson et al. [20], a mean volume of 24 cc by Kallala et al. [19], a maximum volume of 20 cc by Agarwal et al. [23] and 5-30 cc by Menon et al. [21]. McPherson et al. reported that all wound drainage tended to be present when Stimulan with a volume greater than 30 cc was used (Table 1) [20]. However, a statistical correlation between the volume of beads and drainage is yet to be established.

As explained previously, physical phenomena are thought to promote fluid sequestration and trigger inflammation, which is responsible for excessive wound drainage [20,24,25]. This mechanism is thought to be exaggerated in patients with existing medical conditions. McPherson et al. reported that most of their cases with wound drainage were MSIS class B and C cases [20]. Kallala et al. also reported that the rate of complications was greater in patients with medical conditions [19]. More studies are needed to establish and elaborate on this correlation. The rate of wound drainage varies with different calcium sulphate derivatives used and can also be affected by the type of surgery and the indication for intervention. This ranges from 3.6% in a study conducted by Kelly, who used OsteoSet (Wright Medical Technology Inc., Arlington, USA) [26], to 51% as reported by Ziran et al. using calcium sulphate combined with demineralised bone matrix [8].

The use of synthetic calcium sulphate products has offered a potentially promising solution to a problem faced by orthopaedic surgeons worldwide. Over the years, the use of this calcium sulphate antibiotic delivery system has increased. However, clarity regarding the efficacy of this product and its potential side effects is lacking. Side effects such as wound drainage, heterotrophic ossification and hypercalcaemia are well documented [17-23].

## Conclusions

Our study has shown that a small number of patients developed wound leakage after calcium sulphate bead implantation in prosthetic joint surgeries. However, the correlation of these complications with host factors, product volume and placement in surgery is not well understood. More studies are needed to understand this causality and the factors that play a role in complications arising from the use of calcium sulphate beads.

## References

[1] (2020). Joint replacement statistics. https://www.njrcentre.org.uk/njrcentre/Patients/Joint-replacement-statistics.

[2] Williams SN, Wolford ML, Bercovitz A (2015). Hospitalization for total knee replacement among inpatients aged 45 and over: United States, 2000-2010. https://www.cdc.gov/nchs/data/databriefs/db210.pdf.

[3] Wolford ML, Palso K, Bercovitz A (2015). Hospitalization for total hip replacement among inpatients aged 45 and over: United States, 2000-2010. NCHS Data Brief.

[4] Kurtz S, Ong K, Lau E, Mowat F, Halpern M (2007). Projections of primary and revision hip and knee arthroplasty in the United States from 2005 to 2030. J Bone Joint Surg Am.

[5] Springer BD, Cahue S, Etkin CD, Lewallen DG, McGrory BJ (2017). Infection burden in total hip and knee arthroplasties: an international registry-based perspective. Arthroplast Today.

[6] Helgeson MD, Potter BK, Tucker CJ, Frisch HM, Shawen SB (2009). Antibiotic-impregnated calcium sulfate use in combat-related open fractures. Orthopedics.

[7] Cooper JJ, Florance H, McKinnon JL, Laycock PA, Aiken SS (2016). Elution profiles of tobramycin and vancomycin from high-purity calcium sulphate beads incubated in a range of simulated body fluids. J Biomater Appl.

[8] Ziran BH, Smith WR, Morgan SJ (2007). Use of calcium-based demineralized bone matrix/allograft for nonunions and posttraumatic reconstruction of the appendicular skeleton: preliminary results and complications. J Trauma.

[9] Saleh K, Olson M, Resig S (2002). Predictors of wound infection in hip and knee joint replacement: results from a 20 year surveillance program. J Orthop Res.

[10] Ferguson JY, Dudareva M, Riley ND, Stubbs D, Atkins BL, McNally MA (2014). The use of a biodegradable antibiotic-loaded calcium sulphate carrier containing tobramycin for the treatment of chronic osteomyelitis: a series of 195 cases. Bone Joint J.

[11] Beuerlein MJ, McKee MD (2010). Calcium sulfates: what is the evidence?. J Orthop Trauma.

[12] McPherson EJ, Woodson C, Holtom P (2002). Periprosthetic total hip infection: outcomes using a staging system. Clin Orthop Relat Res.

[13] Liberati A, Altman DG, Tetzlaff J (2009). The PRISMA statement for reporting systematic reviews and meta-analyses of studies that evaluate health care interventions: explanation and elaboration. J Clin Epidemiol.

[14] Parvizi J, Zmistowski B, Berbari EF (2011). New definition for periprosthetic joint infection: from the Workgroup of the Musculoskeletal Infection Society. Clin Orthop Relat Res.

[15] Costerton JW, Stewart PS, Greenberg EP (1999). Bacterial biofilms: a common cause of persistent infections. Science.

[16] Kanellakopoulou K, Galanopoulos I, Soranoglou V (2009). Treatment of experimental osteomyelitis caused by methicillin-resistant Staphylococcus aureus with a synthetic carrier of calcium sulphate (Stimulan) releasing moxifloxacin. Int J Antimicrob Agents.

[17] Sandiford NA (2020). Complication rates are low with the use of Stimulan calcium sulphate based antibiotic delivery system in the management of patients with hip-related PJI: early results of a consecutive case series. Hip Int.

[18] Kallala R, Haddad FS (2015). Hypercalcaemia following the use of antibiotic-eluting absorbable calcium sulphate beads in revision arthroplasty for infection. Bone Joint J.

[19] Kallala R, Harris WE, Ibrahim M, Dipane M, McPherson E (2018). Use of Stimulan absorbable calcium sulphate beads in revision lower limb arthroplasty: safety profile and complication rates. Bone Joint Res.

[20] McPherson E, Dipane M, Sherif S (2013). Dissolvable antibiotic beads in treatment of periprosthetic joint infection and revision arthroplasty-the use of synthetic pure calcium sulfate (Stimulan®) impregnated with vancomycin & tobramycin. Reconstructive Review.

[21] Menon A, Soman R, Rodrigues C, Phadke S, Agashe VM (2018). Careful interpretation of the wound status is needed with use of antibiotic impregnated biodegradable synthetic pure calcium sulfate beads: series of 39 cases. J Bone Jt Infect.

[22] Lum ZC, Pereira GC (2018). Local bio-absorbable antibiotic delivery in calcium sulfate beads in hip and knee arthroplasty. J Orthop.

[23] Agarwal S, Healey B (2014). The use of antibiotic impregnated absorbable calcium sulphate beads in management of infected joint replacement prostheses. J Arthrosc Jt Surg.

[24] Lee GH, Khoury JG, Bell JE, Buckwalter JA (2002). Adverse reactions to OsteoSet bone graft substitute: the incidence in a consecutive series. Iowa Orthop J.

[25] Robinson D, Alk D, Sandbank J, Farber R, Halperin N (1999). Inflammatory reactions associated with a calcium sulfate bone substitute. Ann Transplant.

[26] Kelly CM, Wilkins RM, Gitelis S, Hartjen C, Watson JT, Kim PT (2001). The use of a surgical grade calcium sulfate as a bone graft substitute: results of a multicenter trial. Clin Orthop Relat Res.

